# Pharmacokinetics, Tissue Distribution, Excretion and Plasma Protein Binding Studies of Wogonin in Rats

**DOI:** 10.3390/molecules19055538

**Published:** 2014-04-29

**Authors:** Amer Talbi, Di Zhao, Qingwang Liu, Junxiu Li, Ali Fan, Wei Yang, Xing Han, Xijing Chen

**Affiliations:** Center of Drug Metabolism and Pharmacokinetics, China Pharmaceutical University, 24# Tongjiaxiang, Nanjing 210009, Jiangsu, China

**Keywords:** flavonoids, excretion, LC-MS/MS, *Scutellaria baicalensis* Georgi, wogonin

## Abstract

Wogonin is a natural anticancer candidate. The purpose of this study was to explore the pharmacokinetic profiles, tissue distribution, excretion and plasma protein binding of wogonin in Sprague—Dawley rats. A rapid, sensitive, and specific LC-MS/MS method has been developed for the determination of wogonin in different rat biological samples. After i.v. dosing of wogonin at different levels (10, 20 and 40 mg/kg) the elimination half-life was approximately 14 min, the AUC_0-∞_ increased in a dose disproportional manner from 112.13 mg/L·min for 10 mg/kg to 758.19 mg/L·min for 40 mg/kg, indicating a non linear pharmacokinetic profile. After i.g. dosing at 100 mg/kg, plasma levels of wogonin peaked at 28 min with a C_max_ value of 300 ng/mL and a very low oral bioavailability (1.10%). Following i.v. single dose (20 mg/kg), wogonin was detected in all examined tissues (including testis) with the highest levels in kidney and liver. Approximately 21% of the administered dose was excreted as unchanged drug (mainly via non-biliairy fecal route (16.33%). Equilibrium dialysis was used to evaluate plasma protein binding of wogonin at three concentrations (0.1, 0.5 and 2 µg/mL). Results indicated a very high protein binding degree (over 90%), reducing substantially the free fraction of the compound.

## 1. Introduction

**Figure 1:**
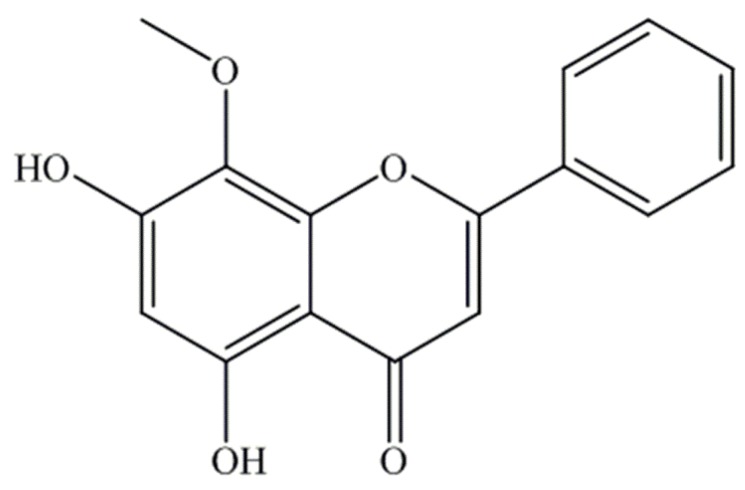
Chemical structure of wogonin.

Wogonin (5,7-dihydroxy-8-methoxyflavone, Figure 1) is one of the major flavonoids extracted from the root of *Scutellaria baicalensis* Gerogi (*Scutellariae radix*), a flowering plant that has long been known as a traditional remedy in East Asian countries [1].

Wogonin has been widely investigated for its antioxidant [2], anti-inflammatory [3,4], and anticancer activities [5,6]. With increasing evidence of its anticancer therapeutic potential and attractive toxicological properties [7,8,9,10,11], wogonin has been recognized as a promising lead compound for new anticancer drug development [12].

Various studies have suggested that wogonin exerts its anticancer effect through modulation of multiple molecular pathways. Wogonin induces apoptosis in hepatocellular carcinoma cells SK-HEP-1 via activation of caspase 3 and alternative expression of p21 protein [13]. Wogonin also can induce apoptosis by activating the AMPK and p53 signaling pathways in human glioblastoma cells [14]. G1cell cycle arrest was induced by wogonin via regulating Wnt/β-catenin signaling pathway and inactivating CDK8 in human colorectal cancer carcinoma cells [15]. Inactivation of GSK-3β and downregulation of ΔNp63 by wogonin enhances apoptosis effect in human nasopharyngeal carcinoma cells through [16].Wogonin inhibits tumor angiogenesis via degradation of HIF-1α protein in four human tumor cell lines, including MCF-7, MDA-MB-231, HepG2, and HCT116 [17]. In addition, wogonin inhibits human oral cancer cells invasion by decreasing the activity of matrix metalloproteinases and urokinase-plasminogen activator [18]. Wogonin has been reported to promote apoptosis and cell cycle arrest in breast cancer through modulation of Akt-dependent signaling pathways [19,20]. Moreover, wogonin exerts its cell apoptosis effect through Ca^+2^ mediated mechanisms [8] and Bax/Bcl-2 pathway [21].

Despite the unceasingly expanding list of postulated targets, *in vitro* to *in vivo* translation of these findings is still poor due to a lack of pharmacokinetic studies. Pharmacokinetics studies of natural compounds play a critical role, not only in the lead druggability assessment and optimization stage, but also in providing clues orienting the design of kinetically relevant models to uncover new targets and molecular pathways underlying these natural agents pharmacological effects [22].

Previous studies dealt with wogonin plasma pharmacokinetics as one of the active agent of traditional remedies administered for the major part orally [23,24,25]. Due to the complexity of these preparations and potential interactions between their different constituents, it’s difficult to fully assess wogonin’s pharmacokinetic behavior. Moreover, to our knowledge, no LC-MS/MS method for the quantification of wogonin in different rat biosamples including plasma, tissue samples, feces, urine and bile has been reported. Therefore, in the present study, an LC–MS/MS method for the determination of wogonin in rat biological matrices was developed and successfully applied to pharmacokinetic, tissue distribution, excretion and plasma protein binding studies of wogonin.

## 2. Results and Discussion

### 2.1. Validation of Analytical Method

The calibration curve of wogonin in rat biological samples was linear, over the range from 4 to 4,000 ng/mL (0, 4, 10, 40, 200, 1,000, 2,000, 4,000 ng/mL) with a lower limit of quantification (LLOQ) of 4 ng/mL. R^2^ for all standard curves were >0.994. The relative standard deviation of precision was 6.6%, 5.4%, and 3.3% at 10, 200, and 2000 ng/mL, respectively. Accuracies determined for intra- and inter-day were all within 100% ± 10% of the actual values. Moreover, no significant matrix effect was observed after dilution. Thus, this optimized LC-MS/MS method has been proved to be sensitive, selective, and rapid for determination of wogonin in rat plasma and different types of tissue samples.

### 2.2. Pharmacokinetics Study

**Figure 2:**
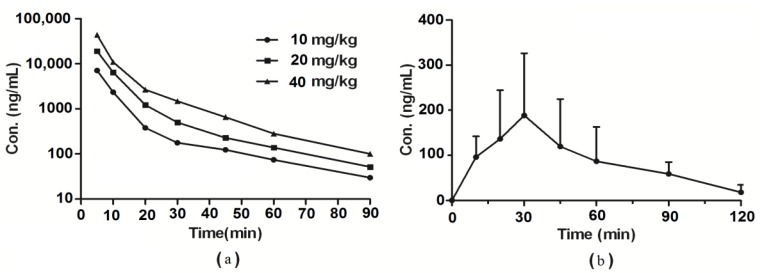
Mean plasma concentration-time profiles of wogonin in rats after receiving a single i.v. dose at different concentration levels (10, 20 and 40 mg/kg wogonin, *n* = 6) (**a**) and plasma concentration-time (mean ± SD) profile of wogonin in rats following i.g. dose of 100 mg/kg wogonin; (**b**) (*n* = 6).

The mean plasma concentration-time profiles are presented in Figure 2, and a summary of the pharmacokinetic parameters of wogonin in rats after intravenous (10, 20 and 40 mg/kg) and intragastric (100 mg/kg) administration is presented in Table 1 and Table 2, respectively. After i.v. administration of wogonin in rats; the elimination half-life was approximately 14 min. AUC_0-∞_ increased in a dose disproportional manner from 112.13 mg/L·min for 10 mg/kg to 758.19 mg/L·min for 40 mg/kg After i.gdosing at 100 mg/kg, plasma levels of wogonin peaked at about 28 min with a Cmax value of 300 ng/mL. Wogonin absolute oral bioavailability was only 1.10%.

**Table 1:** Pharmacokinetic parameters for wogonin in rats after intravenous (i.v.) administration of wogonin at different dose levels (mean ± SD, *n* = 6).

Parameters	Units	i.v.		
		10 mg/kg	20 mg/kg	40 mg/kg
**AUC_0-t_**	mg/L·min	111.14 ± 19.68	286.26 ± 55.21	756.41 ± 26.39
**AUC_0-∞_**	mg/L·min	112.13 ± 18.92	286.75 ± 55.03	758.19 ± 26.91
MRT_0-t_ *	Min	6.96 ± 4.1	6.23 ± 2.81	6.31 ± 0.7
**MRT_0-∞_**	Min	8.15 ± 5.72	6.43 ± 2.94	6.55 ± 0.71
**t_1/2z_**	Min	14.13 ± 4.22	14.58 ± 5.27	13.44 ± 3.55
CLz **	L/min/kg	0.09 ± 0.02	0.07 ± 0.01	0.05 ± 0.01
Vz ***	L/kg	2.9 ± 1.61	1.52 ± 0.96	1.03 ± 0.27
**C_max_**	mg/L	7.12 ± 1.22	19.13 ± 3.06	43.8 ± 6.14

* Mean Residence Time; ** Clearance; *** Volume of distribution.

**Table 2:** Pharmacokinetic parameters for wogonin in rats after intragastric (i.g.) administration of 100 mg/kg wogonin (mean ± SD, *n* = 6).

Parameters	Units	i.g.
		100 mg/kg
AUC_0-t_	mg/L·min	15.84 ± 2.03
AUC_0-∞_	mg/L·min	17.02 ± 2.12
t_1/2z_	Min	27.97 ± 4.73
T_max_	Min	28.33 ± 4.08
CLz/F *	L/min/kg	5.97 ± 0.87
Vz/F **	L/kg	241.9 ± 60.2
C_max_	mg/L	0.3 ± 0.08
F	(%)	1.1 ± 0.18

* Clearance relative to oral bioavailability; ** Volume of distribution relative to oral bioavailability.

### 2.3. Tissue Distribution Study

**Figure 3:**
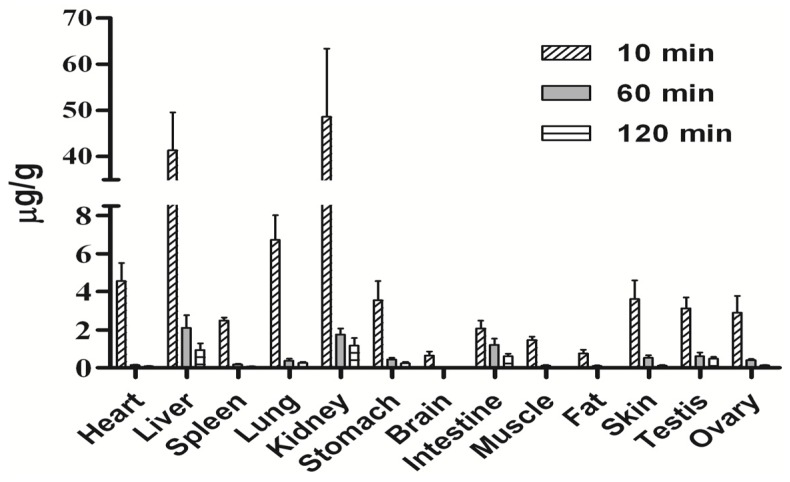
Concentration of wogonin in rat tissues at 10, 60 and 120 min after receiving a single i.v. dose of wogonin at 20 mg/kg. (mean ± SD, *n* = 6, except for testis/ovaries where *n* = 3).

**Figure 4:**
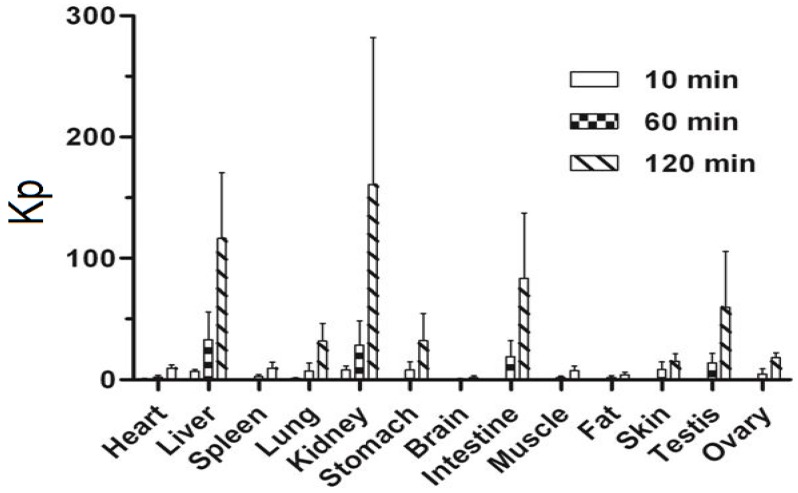
Ratio of tissue toplasma concentrations (Kp) at 10, 60 and 120 min after receiving a single i.v. dose of wogonin at 20 mg/kg. (mean ± SD, *n* = 6, except for testis/ovaries where *n* = 3).

Tissue distribution was assessed at three different time points (10, 60 and 120 min) after intravenous administration of 20 mg/kg wogonin in rats and the results are presented schematically in Figure 3. The results demonstrated that wogonin underwent a rapid and wide distribution to all the examined tissues. Levels of wogonin in kidney and liver were markedly higher than other tissues. It can be worth noticing the presence of wogonin in testis at levels close to those detected in heart, stomach and spleen tissues indicating that wogonin could cross effectively the blood- testis barrier (BTB). The distribution process of wogonin to tissues was very fast, and the mean tissue to plasma ratios (Kp) of wogonin in rats after intravenous administration were increased with time as shown in Figure 4.

### 2.4. Excretion Studies

**Figure 5:**
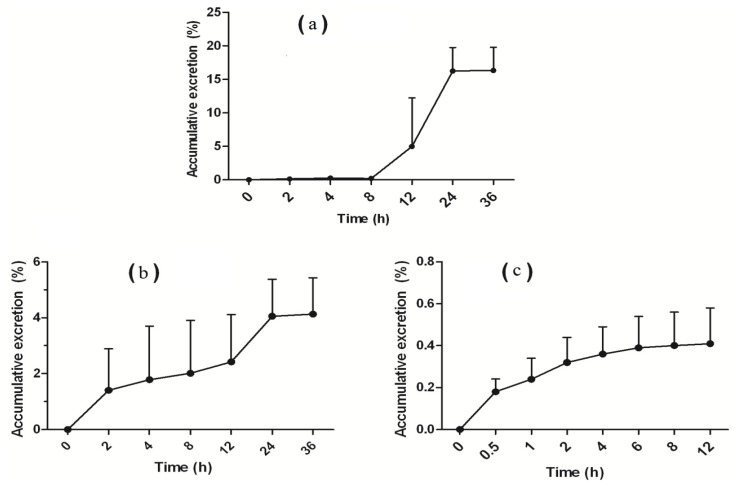
Cumulative percentage of dose excreted in feces (**a**), urine (**b**) and bile (**c**) after intravenous administration of 20 mg/kg wogonin. (mean ± SD, *n* = 6).

The cumulative excretion of wogonin in feces, urine and bile after a single i.v. dose administration (20 mg/kg) was investigated. Excretion data of wogonin in feces, urine and bile (Figure 5) indicated that merely 21% (16.33% ± 3.46%, 4.13% ± 1.30% and 0.41% ± 0.17%, respectively) of the dose administered was excreted as unconverted form.

### 2.5. Protein Binding Study

Equilibrium dialysis is considered a reference method for assessing plasma protein-drug binding rate where nonspecific binding has no impact on the determination of free fraction [26]. Protein binding degrees evaluated using three concentrations levels (0.1, 0.5 and 2.0 µg/mL) of wogonin in rat plasma were 90.26% ± 1.72%, 94.03% ± 2.12% and 90.42% ± 5.41%, respectively.

### 2.6. Discussion

Solid pharmacokinetics knowledge of natural compounds with elusive targets can play a critical role in proper design and choice of *in vitro* pharmacological models to evaluate the molecular mechanisms in a kinetically relevant manner [22,27,28]. In this paper, a rapid, sensitive, and specific LC-MS/MS method for the determination of wogonin in rats plasma, different types of tissues, feces, urine and bile has been reported. This method was satisfactory enough for the study of plasma pharmacokinetics, tissue distribution and protein binding of wogonin in rats and could be applied with modifications for detailed pharmacokinetic studies of other flavonoids. Doses in this study were selected based on previous toxicological studies using Sprague–Dawley rats (NOAEL = 40 mg/kg, intravenously) [9,11].

A non-compartmental method was used to calculate pharmacokinetic parameters of wogonin. After a single i.v. dose, wogonin was rapidly eliminated from plasma. Pharmacokinetic behavior of wogonin appears to be nonlinear with disproportional increases in systemic exposure following an increase in dose. This behavior could be explained by the saturation of some clearance pathways of wogonin at high doses reflected by an approximately 50% decrease in CL(probably due to saturation of UDP-glucuronosyltransferases (UGT) [29], enzymes responsible for wogonin metabolism [30]), which disproportionally increases wogonin plasma concentrations, resulting in a dose-disproportional increase of AUC_0–∞_.

The observed low oral bioavailability of wogonin (1.10%) could be resulting of the compound low solubility [31], and high intestinal and hepatic first pass effect [30,32]. This research also investigated the tissue distribution of wogonin in rats after receiving a single i.v. dose. Results indicated that distribution process was very quick reaching all the examined tissues with the highest levels detected in kidney suggesting a potential role of this organ in the extra-hepatic clearance of wogonin. Moreover, the observed high hepatic levels are in accordance with previous metabolism studies reporting that wogonin undergoes extensive glucuronidation, a Phase II metabolism reaction occurring mainly in liver, with wogonin7β-d-glucuronide as its major metabolite. wogonin7β-d-glucuronide plasma levels were found to be 100-fold higher than the uncoverted molecule [30]. A certain presence of wogonin in brain can be noticed. However, due to low wogonin level and Kp in brain, blood contamination cannot be ruled out. Level of wogonin in testis was close to that in heart, stomach, and spleen, showing that wogonin could effectively cross the Blood-Testis Barrier (BTB). This finding implies that wogonin may be helpful in the treatment of the continuously increasing testis cancer [33].

The main route of excretion of the unconverted form of wogonin was in the feces with a cumulative excreted dose of about 16% and a very low biliairy cumulative dose (0.41%), indicating that wogonin underwent important intestinal excretion. Approximately 80% of the injected dose remains uncounted for. Taken together with metabolism results, these findings suggest that wogonin is mainly excreted as its conjugated form. Further excretion studies exploring wogonin, its metabolite and the potential influence of the intestinal content are needed to fully ascertain wogonin excretion profile. Protein binding ability of a drug in blood plasma is a very important factor influencing its free concentration in blood and tissue distribution. Wogonin showed a high protein binding degree (over 90%) to rat plasma, reducing substancially the free exposure to the compound.

Efficacy of a drug is exerted when achieving appropriate exposure level and time surrounding its target(s) [22]. After i.v. dosing at the highest concentration, wogonin plasma concentration went under 100 µM in about 7 min, under10 µM in about 20 min and under 1 µM in 60 min, with a low unbound fraction (≤10%). Kp values were provided for different tissues at different time points. The data provided in this work should be taken in consideration to select appropriate dosing for *in vitro* pharmacological studies, depending on the explored potential target.

## 3. Experimental

### 3.1. Chemicals and Reagents

Wogonin (>99.0%, purity) was kindly provided by the Department of Medicinal Chemistry, China Pharmaceutical University (Nanjing, China). Repaglinide (>98.0%, purity), wogonin internal standards (IS), was supplied by the National Institute for the Control of Pharmaceutical and Biological Products (Beijing, China). HPLC grade methanol was obtained from Tedia Company (Fairfield, CA, USA). HPLC grade ammonium acetate was purchased from Nanjing Chemical Reagent Co., Ltd. (Nanjing, China). Deionized water was purified using a Milli-Q system (Millipore, Milford, MA, USA). All other reagents were of analytical grade and obtained from conventional commercial sources.

### 3.2. Animal Experimentation

Sprague-Dawley rats (200 ± 20 g) were provided by the Experimental Animal Center of Zhejiang province (Hangzhou, China). Animals were housed under controlled conditions (temperature 22 ± 2 °C, humidity 50% ± 5%, 12 h dark/light cycle) with standardized diet and acclimatized for 7 days, prior to the experiments. The rats were fasted 12 h before the administration of wogonin but with free access to water. The study protocol was approved by the Animal Ethics Committee of China Pharmaceutical University (Nanjing, China).

### 3.3. LC-MS/MS Analysis

The LC-MS/MS system consisted of a SHIMADZU LC-20AD series HPLC system (Shimadzu, Kyoto, Japan) and a Thermo Scientific TSQ Quantum MS/MS system (Thermo Fisher Scientific, Waltham, MA, USA) with electrospray ionization (ESI) source. Data were analyzed by Xcalibur 2.0 software (Thermo Fisher Scientific). Separation was carried out on Hedera ODS-2 column (2.1 × 150 mm, 5 μm, Hanbon, Jiangsu, China) kept at 40 °C. The mobile phase consisted of methanol (mobile phase A) and water (containing 5 mmol/L ammonium acetate, mobile phase B) with rate set at 0.3 mL/min. The gradient elution curve included 0–1.5 min, 45% B; a linear decrease to 5% B within 0.5 min; 5% B for 3.5 min; a linear increase to 45% B within 0.3 min. 45% B for 3.2 min; 9.00 min, stop.

LC-MS/MS was performed in the positive and selected reaction monitoring (SRM) mode. Detected ions were at *m*/*z* 285.1 → 270.1 for wogonin and *m*/*z* 453.0 → 230.0 for the internal standard (repaglinide). The parameters of mass spectrometer were optimized as follows: spray voltage 4.0 kV; capillary temperature 350 °C; sheath gas (N_2_) 35 Arb; auxiliary gas (N_2_) 5 Arb and collision gas (Ar) 1.0 mTorr.

### 3.4. Pharmacokinetic Studies

Twenty four rats were randomly assigned to four groups, each six rats (three male, three female), and received a single intravenous administration of wogonin at 10, 20 and 40 mg/kg, respectively, or intragastric administration of 100 mg/kg. After dosage, blood samples were collected into heparinized tubes at 5, 10, 20, 30, 45, 60 and 120 min for the i.v. groups, and at 10, 20, 30, 45, 60, 90 and 120 min for the i.g. group. Plasma was isolated from the blood samples by centrifugation and then stored at −70 °C until analysis.

Plasma samples (100 μL) were spiked with 20 μL internal standard (repaglinide, 200 ng/mL) and extracted with 600 μL of ethyl acetate by vortexing for 10 min. The organic and aqueous phases were separated by centrifugation at 12,000 g for 3 min. The upper organic phase was transferred to another tube and evaporated to dryness at 50 °C.The residue was dissolved in 100 μL acetonitrile and vortexed. A 10 μL aliquot of the solution was injected onto the LC-MS/MS system for analysis.

### 3.5. Tissue Distribution Study

Another group of eighteen rats were i.v. administered a single dose of 20 mg/kg wogonin via tail vein. Tissues (heart, liver, spleen, lung, kidney, stomach, brain, small intestine, testis/ovaries, skin, muscle and fat) were promptly harvested at 10 or 60 or 120 min after dosing (six rats per time point, three male and three female) and thoroughly rinsed in ice-cold saline to eliminate blood and other content. Blood samples were collected into heparinized tubes before tissue excision. Each tissue sample was homogenized (Tissue sample:saline ratio of 1:3, w/v). Tissue samples were stored at −70 °C until further analysis. The preparation process for analysis was the same as described above for plasma.

### 3.6. Excretion Studies

Six rats (three male and three female) were housed in separate metabolic cages and received a single intravenous dose of wogonin at 20 mg/kg via the tail vein. The urine and feces samples were collected at 0–2 h, 2–4 h, 4–8 h, 8–12 h, 12–24 h, and 24–36 h post dosing. Another six rats (three male, three female) were i.v. dosed at 20 mg/kg wogonin after bile duct cannulation. The bile samples were collected at 0.0–0.5 h, 0.5–1 h, 1–2 h, 2–4 h, 4–6 h, 6–8 h, and 8–12 h intervals. Feces were weighed and homogenized (feces:water ratio of 1:4, w/v) and the volume of urine and bile samples was measured before storage at −70 °C. The samples were then subjected to the same procedure as described for the plasma samples.

### 3.7. Plasma Protein Binding Test

A modified equilibrium dialysis method [34] was applied in this study to determine the plasma protein binding of wogonin in rat plasma. Outside the bag filter was phosphate buffer (0.02 mol/L, pH 7.40) mixed with NaCl (0.15 mol/L), while inside the bag filter was rat plasma (500 μL). Three groups (*n* = 3) were prepared with the concentrations of wogonin outside the bag filter set at 0.1, 0.5 and 2 µg/mL, respectively. The dialysis system was incubated at 4 °C for 72 h to achieve equilibrium. The resulting plasma and buffer dialysates were promptly recovered and analyzed by LC–MS/MS after sample preparation as described above. The binding degree of wogonin in the equilibrium dialysis experiments is expressed according to the equation of:

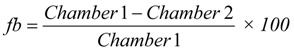
(1)
where *fb* is the bounded fraction, Chamber 1 is the compound concentration in the plasma compartment and Chamber 2 is the concentration of the compound in the phosphate buffer compartment.

### 3.8. Pharmacokinetic and Statistical Analysis

The pharmacokinetic parameters were calculated using the pharmacokinetic software DAS 2.1.1 (Mathematical Pharmacology Professional Committee of China) by non-compartmental method. The elimination half-life (t _1/2_) was determined by linear regression of the terminal portion (last three points) of the plasma concentration-time curve. The area under the plasma concentration-time curve from zero to the last measurable plasma concentration point (AUC_0-t_) was calculated by the linear trapezoidal method. Extrapolation from time zero to infinity (AUC_0-∞)_ was calculated as follows:

AUC_0-∞_ = AUC_0-t_ + *C*_t_/*k*_e_ (2)

where *C*t was the last measurable plasma concentration and *k*e was the terminal elimination rate constant. Absolute oral bioavailability (*F*) of wogonin was calculated according to following the equation:

*F* (%) = (AUC _ig_ × dose_iv_)/(AUC _iv_ × dose_ig_) × 100 (3)

where AUC_ig_ and AUC_iv_ are the AUC values after intragastric and intravenous administration of wogonin, respectively, and D_iv_ and D_ig_ are the doses administered for intravenous and intragastric administration of wogonin, respectively. Data were presented as means with their standard deviation (mean ± SD).

## 4. Conclusions

In this study, a fully validated LC-MS/MS method was successfully applied to investigate the pharmacokinetic characteristics of wogonin in rats, including plasma kinetics, tissue distribution, excretion and plasma protein binding properties. Beyond the increased knowledge of pharmacokinetic profiles for wogonin, the present study would contribute to further research.
